# Seroprevalence of Hepatitis E Virus in Roma Settlements: A Comparison with the General Population in Slovakia

**DOI:** 10.3390/ijerph15050904

**Published:** 2018-05-03

**Authors:** Monika Halánová, Eduard Veseliny, Zuzana Kalinová, Peter Jarčuška, Martin Janičko, Ingrid Urbančíková, Daniel Pella, Sylvia Dražilová, Ingrid Babinská, HepaMeta Team

**Affiliations:** 1 Department of Epidemiology, Faculty of Medicine, Pavol Jozef Šafárik University in Košice, 04180 Košice, Slovakia; monika.halanova@upjs.sk (M.H.); zuzana.kalinova@upjs.sk (Z.K.); ingrid.babinska@upjs.sk (I.B.); 2 1st Department of Internal Medicine, Faculty of Medicine, Pavol Jozef Safarik University in Kosice, 04011 Košice, Slovakia; peter.jarcuska@upjs.sk (P.J.); martin.janicko@gmail.com (M.J.); daniel.pella@upjs.sk (D.P.); 3 Department of Pediatrics, Faculty of Medicine, Pavol Jozef Šafárik University in Košice, 04011 Košice, Slovakia; ingrid.urbancikova@dfnkosice.sk; 4 Department of Internal Medicine, University Hospital Poprad, 05801 Poprad, Slovakia; drazilova.s@nemocnicapp.sk

**Keywords:** hepatitis E, prevalence, risk factors, Roma population, Slovakia

## Abstract

Hepatitis E infection is one of the most frequent causes of acute hepatitis in the world. Currently five human genotypes with different geographical distributions and distinct epidemiologic patterns are identified. In Slovakia, only rare cases of hepatitis E have been reported in past years. Because the most important risk factors associated with HEV infection include consumption of contaminated pork meat and poor hygienic standards, the aim of the study was to evaluate the prevalence of anti-HEV total antibodies and the main risk factors for HEV in the population living in separated and segregated Roma settlements (*n* = 195), which represent places with increased risk of infection in Slovakia and to compare it with the prevalence in the general population (*n* = 69). Of 264 respondents included in the study, 47 (17.8%) showed positivity for anti-HEV antibodies, 42 of whom were Roma (21.5%, *n* = 195) and 5 (7.2%, *n* = 69) non-Roma. The population living in Roma settlements lives in poorer conditions and are at higher risk of HEV in comparison to the general population. However, differences in living conditions within the settlements do not contributed to lower risk of HEV antibody prevalence between Roma living in settlements.

## 1. Introduction

Hepatitis E infections, belonging to a group of emerging diseases, are caused by a single-stranded, positive-sense RNA hepatitis E virus (HEV) of the *Hepeviridae* family [1,2]. This family is divided into the two genera, *Orthohepevirus* which currently contains four species (*Orthohepevirus A* to *D*) and *Piscihepevirus* with one species (*Piscihepevirus A*)*.* Isolates of HEV infecting humans belong to the *Orthohepevirus A* species [3]. At present, eight genotypes (HEV1–HEV8) have been identified based on the nucleotide sequences of the genome in this species, the first four of which (HEV1–HEV4) and HEV7 are recognised in the human population [4]. The genotypes differ from each other by geographical distribution and distinct epidemiological and disease patterns, including sources of infection. Genotypes HEV1 and HEV2 are mainly transmitted by the faecal-oral route via contaminated water and they are responsible for the majority of human infections in endemic tropical and subtropical countries with strictly human sources of infection [5]. However, genotypes HEV3, HEV4 and HEV7 are zoonotic and can be detect in human as well as different species of animals in Europe, Asia and the Americas [6,7]. Domestic pigs and wild boar represent the most important sources of infection for human [8,9]. These genotypes are mainly spread via consumption of raw meat of infected animals or via vegetables and fruits washed with contaminated water. Other ways of transmission are less frequent and include vertical transmission, transmission by direct contact and parenteral transmission [10,11,12,13].

Estimated worldwide incidence of hepatitis E is about three million human infections per year resulting in about seventy thousand deaths [14]. About two billion people live in areas with a high risk of HEV infection [15,16].

The incubation period of HEV infection is estimated to be between two and six weeks (up to 60 days) [17]. Infection in humans is mostly asymptomatic, without any clinical symptoms. Also, acute infection is usually mild and self-limiting, but it can become chronic in immunocompromised patients. In pregnant women, HEV infection might lead to fulminant hepatitis with a fatality rate of up to 30% [18].

Over the last 10 years, a 10-fold increase in reported cases has been observed in EU/EEA countries, from 514 cases in 2005 to 5617 cases in 2015 [19]. Several EU/EEA countries have reported an apparent increase in human cases related to HEV infection in recent years, which might be associated with better HEV detection and diagnosis as well as an increasing awareness among clinicians [19,20].

In Slovakia, there is no specific law establishing systematic screening for this infection, and only rare cases of imported hepatitis E have been reported in past years. The first cases of autochthonous hepatitis E were reported in 2013, and to date only 83 autochthonous cases have been reported [21].

Because the most important risk factors associated with HEV infection include consumption of contaminated pork meat and poor hygienic standards, the aim of the study was to evaluate the prevalence of anti-HEV total antibodies and the main risk factors for HEV in the population living in separated and segregated Roma settlements, which represent in Slovakia the places with increased risk of infection.

## 2. Materials and Methods

### 2.1. Study Population

Data from the cross-sectional population-based Hepa-Meta study conducted in Eastern Slovakia during 2011 were used. The aim of this study was to detect the prevalence of viral hepatis diseases, metabolic syndrome and selected bacterial and parasitic infectious diseases in the Roma population living in separated and segregated settlements and to compare the obtained results with the occurrence in the general population. The methodology of this study was previously described in detail by Madarasová Gecková et al. [22]. The studied population involved inhabitants of 10 different segregated Roma communities in Eastern Slovakia. The non-Roma citizens from the general population from the same region were used as a control group. 

The final sample examined the presence of anti-HEV total antibodies comprised of 264 people aged 18–55, 195 of whom were living in Roma settlements (mean age = 34.8; SD = 8.7; 34.9 men) and 69 coming from the general population (mean age = 34.0; SD = 8.3; 47.8 men) (Table 1).

Data were collected via analysed blood and a questionnaire. Collection of venous blood was done under standard conditions from a peripheral vein in the antecubital fossa. After centrifugation of the blood, serum samples were collected and stored at −20 °C until their use in the serological test.

The questionnaire was prepared by a group of experts made up of Roma health mediators, community workers, public health experts and academics. It gathered information about sociodemographic background, living conditions, health-related behaviour, health and health care use. Employment status was measured by asking respondents the question: “Are you currently employed or unemployed?” The number of people per house was determined through the question: “How many people live in the household?” Highest education was measured by asking respondents the question: “What is your highest educational degree attained?” Possible responses were: Unfinished elementary/Finished elementary/Apprenticeship/Secondary/University. We merged the first two categories into one category (Elementary) and last two categories into one category (Higher education). Standard household equipment was examined by questioning the respondents: “Is there a… in your house?” Possible answers were: Functioning sewage system/Water supply/Functional flushing toilet/Functional bathroom or shower/Electricity supply.

### 2.2. Laboratory Examination

Detection of anti-HEV total antibodies was done using a commercial enzyme-linked immunosorbent assay (ELISA) kit (DRG Instruments GmbH, Marburg, Germany), according to the manufacturer’s instructions. This is a qualitative assay for detection of total HEV antibodies (mostly IgG, IgM and IgA) in serum. 

### 2.3. Statistical Analyses

Basic descriptive statistics were used for analysis of the obtained results. Relative risks (RR) and their 95 percent confidence intervals (95% CI) were estimated for the occurrence of anti-HEV total antibodies. The association between gender, living conditions and the prevalence of anti-HEV total antibodies were tested using the chi-square tests. The analyses were performed with SPSS Statistics version 20 (IBM, Armonk, NY, USA).

### 2.4. Ethical Approval

The study was performed in accordance with the principles of the Declaration of Helsinki and approved by the Ethics Committee of the Faculty of Medicine at P. J. Šafárik University in Košice (No. 104/2011). Participation in the study was fully voluntary and anonymous, and written informed consent was obtained from each person prior to the medical examination.

## 3. Results

### 3.1. Basic Characteristics of Study Population

Analysis of basic characteristics of the people living in segregated settlements and those from the general population showed significant differences in unemployment (87.7% vs. 27.5%, *p* < 0.0001), number of people per house (7.1 vs. 4.5, *p* < 0.0001) and education (78.5% vs. 1.4%, *p* < 0.0001) (Table 1). 

### 3.2. Seroprevalence of Hepatitis E

Of the 264 respondents included in the study, 47 (17.8%) showed positivity for anti-HEV antibodies, 42 of whom were Roma (21.5%, *n* = 195) and five (7.2%, *n* = 69) non-Roma. The highest positivity was detected in Roma men (29.4%). Upon comparing the relative risk of anti-HEV antibodies occurrence in the Roma and non-Roma groups, we found the risk of infection to be nearly 3-time higher in the people living in segregated Roma settlements than in the group of people from the general population. This risk was very similar when comparing the incidence according to gender; 3.2-time higher for Roma men, resp. 3.1-timeshigher for Roma women (Table 2). 

### 3.3. Social and Living Conditions Factors Associated with Hepatitis E

The influence of basic household facilities, such as a sewage system, water supply, flush toilet, bathroom or shower, and electricity supply were analysed in both Roma and non-Roma (Table 3) and also separately for respondents living in Roma settlements in relation to HEV seropositivity (Table 4). 

As one may see in Table 3, persons living in Roma settlements reported significantly less frequently access to sewage systems, water supplies, flush toilets, bathrooms or showers, and electricity supply. 

Comparison within Roma settlements revealed that access to household facilities does not decrease risk of HEV seroprevalence. Roma having and not having access to sewage system, water supply, flush toilet, bathroom or shower, and electricity supply do not differ in risk of HEV seroprevalence (see Table 4). 

## 4. Discussion

Communicable diseases are increasingly threatening public health. The risk of the emergence and spread of communicable diseases significantly increases with travel, trafficking, legal and illegal migration of people, as well as unfavourable socio-economic conditions and low standard of living [23]. In Slovakia, this category mainly includes citizens living in segregated and separated Roma settlements.

Slovakia belongs demographically among countries with the highest percentage of Roma. In the last census, according to the Statistical Office of the Slovak Republic, 106,583 citizens from the total population of 5,410,728 were officially registered for Roma nationality, which represents only 2%; however, the estimated number of Roma citizens in Slovakia ranges from 320,000 to 500,000, with a percentage of the total population between 8% and 10% [24,25].

The Roma in Slovakia do not live in a coherent territory but rather are dispersed throughout the country, and there are large differences in their number and concentration in the individual regions of the Slovakia.

Geographically, the highest number of Roma (about 60%) lives in eastern Slovakia, most of them in segregated and separated settlements located on the outskirts of municipalities or towns, respectively [24]. The health status of minority populations is according to several references worse than the general population, and is affected by serious social problems, such as poverty, low income, unemployment, low education and inappropriate housing. In general, there is a higher prevalence of infectious diseases, injuries, poisoning and burns in this group. In the field of infectious diseases, they are a risk group for tuberculosis, viral hepatitis and parasitic diseases, but unfavourable social and economic conditions affect the incidence of other communicable diseases.

In our study, we focused on monitoring hepatitis E, which has worldwide distribution and globally is a cause of morbidity and mortality [15]. For many years, hepatitis E was considered to be a disease occurring only in poor developing countries, but in recent years, the situation has changed dramatically, and it is evident that hepatitis E is a threat to global health [26]. Outbreaks of infections are observed mainly in tropical and subtropical countries with low hygiene and sanitary conditions, but sporadic cases of HEV infection have been described in developed countries, too. The fact that the virus can be spread by the faecal-oral route suggests that infection may occur in places where there are poor water supplies and inadequate sewage and waste disposal. Another important factor that may affect the occurrence of HEV infection in humans is the presence of infected animals. In Europe, domestic pigs and wild boar are important sources of zoonotic transmission of genotypes HEV-3 and HEV-4, and infection is mainly transmitted after consumption of contaminated and improperly cooked pork or other pork or game products. 

In Slovakia, infection was detected for the first time in animals only in 2017, when Jackova et al., published on the prevalence of hepatitis E virus in the domestic pigs. RT-PCR analysis of 269 enteric samples revealed that 32 pigs (11.9%) of all age categories were HEV RNA positive [27]. Moreover, to date only 83 cases of autochthonous disease in humans have been officially reported. This number is much less in comparison with, for example, the neighbouring Czech Republic, where 412 cases were reported in 2015 and 339 in 2016, and the estimated seroprevalence is 12.8%. Generally, published seroprevalence rates in Europe have ranged from 0.7% (drug users in Italy) to 52% (blood donors in France) depending on the geographical location, type of study cohort and the assay used [28,29,30,31,32,33,34]. 

The seroprevalence of HEV in our sample (21.5% in people living in Roma settlements vs. 7.2% in the general population) corresponds with the findings of these studies but indicates a significant difference in incidence between these two monitored groups. The consumption of improperly cooked pork or products from infected pigs (especially liver) is assumed to be the main risk factor of hepatitis E in our conditions, though hygiene conditions may also be closely related to the risk of infection. Although according our study most people living in Roma settlements usually have no problem with electricity supply (81.5%) and can cook their food properly, the lack of basic household facilities, such as a sewage system or drinking water supply (they mostly use water wells or streams) as well as problems with hygiene and sanitation of the environment, can also play important role in cross-contamination when handling food or virus excretion from the faeces of infected people into water supplies. 

The strengths of this study are that we were successful in recruiting a considerable number of respondents from the hard-to-reach Roma population living in settlements. This was achieved by following the principles of community-based participatory research through engaging Roma community workers. Our study also has some limitations. To assess the contribution of Roma ethnicity on seroprevalence of hepatitis E virus should be done with caution. Living conditions in Roma settlement are very specific (restricted or low access to drinking water and sanitation, living in the outskirts of villages in very poor conditions) and it is difficult to find a comparable control group in the general population living in similar conditions [35]. Moreover, Roma living in settlements represent only part of whole Roma population and our findings on higher seroprevalence of HEV in Roma settlements should be attributed rather to very poor living conditions than to the ethnicity itself. On the other hand, having better living conditions might not be reflected in lower risk of HEV due to the value of “sharing amenities” which is deeply embedded in Roma culture [22]. 

## 5. Conclusions

Based on our obtained results, we can conclude that the seroprevalence of viral hepatitis E is significantly higher in people living in separated and segregated Roma settlements than in the general population. Illegal huts, a devastated environment and no access to basic infrastructure, such as tap water, sewage system and waste disposal, has an influence on poor health status and troubles with hygiene and increases the risk of development of many infectious diseases, including hepatitis E. 

## Figures and Tables

**Table 1:** Basic characteristics of the Roma (*n* = 195) and non-Roma (*n* = 69) samples and p-values for differences between the Roma and non-Roma population in eastern Slovakia.

Parameter	Roma *n* (%)	Non-Roma *n* (%)	*p*
Male gender	68 (34.9)	33 (47.8)	n.s. ^a^
Age, *mean* (*STD*)	34.8 (8.7)	34.0 (8.3)	n.s. ^b^
Unemployed	171 (87.7)	19 (27.5)	<0.001 ^a^
Number of people in house, *mean* (*STD*)	7.1 (3.6)	4.5 (1.8)	<0.001 ^b^
Education			
Elementary	153 (78.5)	1 (1.4)	<0.001 ^a,c^
Apprenticeship	34 (17.4)	24 (34.8)	
Higher	7 (3.6)	43 (62.3)	

^a^ chi-square test; ^b^ *t*-test, n.s. not significant; ^c^ *p*-value for Education is across Elementary, Apprenticeship and Higher.

**Table 2:** Prevalence of Hepatitis E infection in Roma (*n* = 195) and non-Roma population (*n* = 69).

Gender	Roma *n* (%)	Non-Roma *n* (%)	Chi-Square Test	Relative Risk (95% CI)
Men	20 (29.4)	3 (9.1)	<0.1	3.23 (1.03–10.11)
Women	22 (17.3)	2 (5.6)	<0.1	3.11 (0.76–12.63)
Σ	42 (21.5)	5 (7.2)	<0.01	2.97 (1.22–7.20)

**Table 3:** Basic household facilities of respondents living in Roma settlements (*n* = 195) and respondents from the general population (*n* = 69).

Parameter	Roma *n* (%)	Non-Roma *n* (%)	*p*
Sewage system	79 (40.5)	56 (81.2)	<0.001 ^a^
Water supply	97 (49.7)	68 (98.6)	<0.001 ^a^
Flush toilet	87 (44.6)	68 (98.6)	<0.001 ^a^
Bathroom or shower	85 (43.6)	68 (98.6)	<0.001 ^a^
Electricity supply	159 (81.5)	68 (98.6)	<0.001 ^a^

^a^ chi-square test.

**Table 4:** Basic household facilities of HEV seropositive respondents living in Roma settlements (*n* = 195).

Parameter	HEVseropositive (*n*/%)	Chi-Square Test
Sewage system		
Yes	17 (21.5)	0.996
No	25 (21.6)	
Water supply		
Yes	18 (18.6)	0.314
No	24 (24.5)	
Flush toilet		
Yes	18 (20.7)	0.796
No	24 (22.2)	
Bathroom or shower		
Yes	17 (20.0)	0.646
No	25 (22.7)	
Electricity supply		
Yes	33 (20.8)	0.576
No	9 (25.0)	
